# Food Supplementation Fails to Reveal a Trade-Off between Incubation and Self-Maintenance in Female House Wrens

**DOI:** 10.1371/journal.pone.0106260

**Published:** 2014-09-03

**Authors:** Cassie J. Lothery, Charles F. Thompson, Megan L. Lawler, Scott K. Sakaluk

**Affiliations:** Behavior, Ecology, Evolution and Systematics Section, School of Biological Sciences, Illinois State University, Normal, Illinois, United States of America; Utrecht University, Netherlands

## Abstract

Incubating birds must allocate their time and energy between maintaining egg temperature and obtaining enough food to meet their own metabolic demands. We tested the hypothesis that female house wrens (*Troglodytes aedon*) face a trade-off between incubation and self-maintenance by providing females with supplemental food during incubation. We predicted that food supplementation would increase the amount of time females devoted to incubating their eggs, lower their baseline plasma corticosterone levels (a measure of chronic stress), and increase their body mass, haematocrit (a measure of anaemia), and reproductive success relative to control females. As predicted, food-supplemented females spent a greater proportion of time incubating their eggs than control females. Contrary to expectation, however, there was no evidence that food supplementation significantly influenced female baseline plasma corticosterone levels, body mass, haematocrit, or reproductive success. However, females with high levels of corticosterone at the beginning of incubation were more likely to abandon their nesting attempt after capture than females with low levels. Corticosterone significantly increased between the early incubation and early nestling stages of the breeding cycle in all females. These results suggest that although food supplementation results in a modest increase in incubation effort, it does not lead to significantly lower levels of chronic stress as reflected in lower baseline corticosterone levels. We conclude that female house wrens that begin the incubation period with low levels of plasma corticosterone can easily meet their own nutritional needs while incubating their eggs, and that any trade-off between incubation and self-feeding does not influence female reproductive success under the conditions at the time of our study.

## Introduction

Organisms have limited resources to allocate among reproduction, growth, and maintenance [1], and the optimal allocation of resources that maximizes an individual's fitness may differ considerably in different environments. In many bird species, males and females share reproductive duties, such as incubation, nestling provisioning, and defence of the nest, which allows each parent to allocate more time to self-maintenance (e.g., foraging) than care-giving parents in those species in which only one parent provides most of the care [2]. However, in many species in which both parents make significant contributions to parental care, these duties are not equally shared. In some species, males never incubate the eggs, rarely feed their mate during the incubation period [3], and vary considerably in the amount of provisioning they provide to their nestlings and fledglings [4]–[6]. In such species, reproductive costs (i.e., negative effects of current reproductive effort on future survival and reproduction; *sensu* [7], [8]) fall disproportionately on the female. Although it has long been assumed that the nestling and fledgling periods demand the greatest reproductive effort and, therefore, exact the highest reproductive costs [9], [10], the costs associated with incubation have begun to receive increased attention [11]–[14]. After all, females must ensure that egg temperatures are maintained within a narrow range optimal for embryonic development while ensuring that they obtain sufficient food for themselves to meet energetic demands.

Because it takes more energy for females to re-warm eggs to the optimal temperature for embryo development after a long foraging bout away from the nest than it does to maintain eggs continuously at their ideal temperature [15], selection should favour females that optimize the length and frequency of foraging bouts during incubation to minimize costs associated with maintaining ideal egg temperatures. Indeed, studies that have manipulated the temperatures of nests and eggs suggest that the need for self-maintenance may play a major role in regulating female behaviour during incubation [16]–[20].

Availability of food is likely the primary factor that limits the amount of time females can spend incubating their eggs [21]. This hypothesis is supported by the results of food supplementation studies showing that supplemented females increase their nest attentiveness compared with controls [5], [22]–[25] and that this leads, in some cases, to shorter incubation periods [26]. By increasing time spent on the nest incubating eggs and shortening the incubation period, females lower the risk of nest predation [27]. Thus, the need to balance incubation demands while obtaining sufficient food to meet their own metabolic demands can result in higher rates of nest predation, poor regulation of egg temperatures, and increased chronic stress, resulting ultimately in lower female survival or lower reproductive success.

The effects of balancing the demands of incubation and self-maintenance behaviour are routinely assessed by taking several different physiological measures, including baseline corticosterone levels in the blood, degree of anaemia (as measured by haematocrit), and body mass. Corticosterone, the major glucocorticoid hormone found in birds, has multiple effects on a variety of organ systems, including the central nervous system and gonads both during ontogeny and later in life (reviewed in [28], [29]). The main action of corticosterone is to modulate the release of glucose in response to both predictable and unpredictable environmental challenges [30], [31], making measurement of baseline plasma corticosterone the hormone of choice when assessing how individuals are coping with their environment [32]–[34]. The concentration of baseline plasma corticosterone increases during chronically stressful periods, such as when food is limited as well as when predators are present [32], [33], [35]. Thus, if females are unable to obtain enough food to maintain a positive energy balance during incubation, plasma corticosterone levels should increase and both haematocrit [32] and body mass should decrease.

In this study, we tested the hypothesis that incubating female house wrens (*Troglodytes aedon*) engage in a trade-off between the time invested in incubation behaviour and self-maintenance that is mediated by their need to meet their own energetic demands. If food availability influences how females allocate their resources between reproduction and self-maintenance, we predicted that females receiving a food supplement during incubation would: (1) spend more time incubating their eggs; (2) have shorter incubation periods; (3) have lower baseline corticosterone levels; (4) be in better condition, as reflected in body mass and haematocrit; and (5) have higher reproductive success than control females.

## Methods

### Ethics Statement

The ParkLands Foundation (Merwin Preserve) and the Sears and Butler families provided access to their properties for this study. This project was approved by the Illinois State University Institutional Animal Care and Use Committee (Protocol No. 05-2010), and conducted in accordance with the United States Geological Survey banding permit 09211 and U.S. Fish & Wildlife Service collecting permit MB692148-0. Data used in analyses can be obtained from the Dryad (http://datadryad.org/) repository.

### Study species, study site, and nest-monitoring

House wrens are small (10–12 g), insectivorous song birds that breed throughout the mid-latitudes of North America. They return from their wintering grounds to breed in central Illinois from late April to September (see Figure 3 in [36]). Only female house wrens incubate the eggs and brood the nestlings, but both adults provision nestlings and fledglings. Males only rarely engage in mate feeding (i.e., provisioning the female while she is incubating [3], [36]). Nests are constructed in preformed cavities, such as tree holes, so house wrens readily accept artificial nestboxes as nesting sites. House wrens are tolerant of human disturbance around their nest site, and readily eat insects provided to them as a supplement to their normal diet [24], [37].

This study took place at the Mackinaw study area in McLean County, Illinois, (40°40′N, 88°53′W; elevation 220 m) during the first brood (May-June) of the 2011 breeding season. The study area contains 700 nestboxes placed 30 m apart in north-south transects spaced 60 m apart (see [38]). The Mackinaw River bisects the study area, which consists of upland and bottomland secondary-growth deciduous forest. The experiment took place in the northeastern region of the study area, a tract of closed-canopy upland and floodplain forest areas (see Figure 1 in [38]).

**Figure 1:**
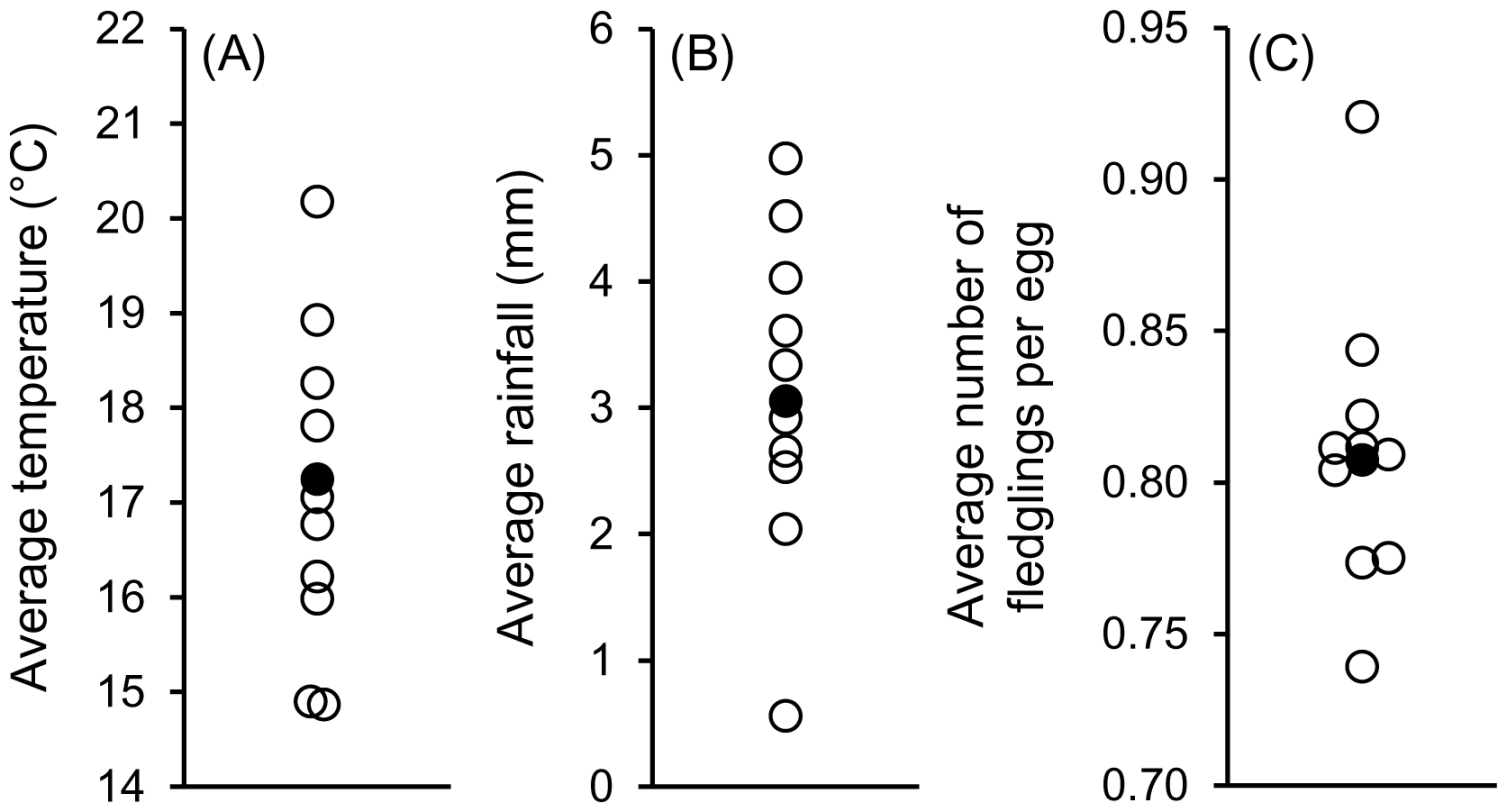
Average daily temperature (A), rainfall (B), and number of fledglings produced per egg laid (C) during May of the ten years preceding the study (2001–2010, open circles) and May of the year of the study (2011; filled circle).

Nestboxes are fixed to a 1.5-m metal pole and rest atop a 48.3-cm diameter aluminium disk that serves to deter nest predators (for nestbox dimensions and construction materials, see [39]). Each nestbox has a permanently mounted, sliding metal door that is used to trap adults in the box.

To determine how temperature and rainfall during May (the time of year when clutches of the first brood are being incubated) in 2011 compared with those during May in the preceding 10 breeding seasons (2001–2010), we obtained data from the National Data Center's Chenoa, McLean County, Illinois, weather station located ≈16 km northeast of the study area; we also compared a measure of first-brood reproductive success (average number of fledglings produced per egg) in those years with that in 2011. For all three measures, 2011 fell in the middle of the range (Fig. 1).

Nestboxes were checked twice weekly for evidence of nest-building activity and egg-laying. Nests were monitored regularly to determine clutch size (number of eggs laid), hatching success (number of eggs hatching), and fledging success (number of nestlings surviving to leave the nest).

### Food supplementation

During incubation, from the second day after egg-laying ended and the eggs were warm to the touch (incubation-day 2; incubation-day 0 is the day the last egg was laid), to the hatching of the first egg (brood-day 0), female house wrens were given a daily food supplement of 15 g of live mealworms (*Tenebrio molitor*) topped, for dietary variety, with 5–10 freshly freeze-killed crickets (*Gryllodes sigillatus* or *Acheta domesticus*), of which ≈90% were *G. sigillatus*. The quantity of mealworms used was based on the estimate that a 10.6-g adult house wren expends approximately 61 kJ/day during the nestling stage [40], and that mealworms contain 11.59 kJ/g [37]. As another passerine species has an assimilation efficiency for mealworms of 0.65 [41], an adult house wren would need to consume about 8.2 g of mealworms to satisfy its daily energy requirements. Thus, 15 g of mealworms represents approximately 185% of the daily energetic requirement of an adult that is provisioning nestlings and, therefore, greatly surpasses a female's energy requirements during the incubation stage [42].

A treatment (supplement or control) was randomly assigned to the first nest at which incubation had begun, and then in alternating fashion as each new nest entered the incubation period. Nestboxes in both treatments had a 30-mm-diameter x 50-mm-deep plastic film canister tacked securely to the interior next to the entrance, into which the food was placed in the supplement treatment. Nest watches were conducted using digital video recordings to confirm that females were consuming the food supplement, as they frequently brought mealworms outside of the nestbox to consume them while perched on top of the nestbox or the predator guard. To ensure that the adults were habituated to the camera, all video observations (about 1 h in length) were recorded 24 h after a dummy camera had been placed 1.5–2 m from the nestbox. Start times of video observations were randomly assigned between 07:00 and 11:00 Central Daylight Time (CDT). The majority of females consumed the entire food supplement each day. During recordings, no male brought food to the incubating female or entered the nestbox.

### Incubation behaviour

When the female is inside the nestbox after incubation has commenced, she is applying heat to the eggs because eggs are never cold to the touch when a female is flushed from the nestbox (unpubl. data). Female incubation behaviour (presence or absence in the nestbox) was measured using digital video recordings (about 1 h in length; *n*_con_ = 18, *n*_exp_ = 21). The same protocol was used for video observations during incubation as described above (see Food Supplementation section). Videos were recorded once on incubation-day 7–9 for each nest in both treatment groups. A few food-supplemented nests were not included in the final analysis because of disturbance during the video recording or camera malfunction; these missing observations are not included in the sample sizes listed above. Four measures of female incubation behaviour were determined from the digital video recordings: (1) incubation constancy (the proportion of time a female spent on the nest during the recording period), (2) on-bout length (mean length of time spent inside the nestbox), (3) off-bout length (mean length of time spent outside the nestbox), and (4) bout frequency (the number of off-bouts/h) (see [19], [24]).

### Capturing females and collecting blood samples

A blood sample (≈50 µL) was obtained from females before food supplementation began (incubation-day 2) and a second was taken post-supplementation (brood-day 1). Blood samples were obtained by using a sterile lancet to pierce the left brachial vein after swabbing the surface with alcohol. In the field, the blood was collected in heparinized microcapillary tubes that, prior to returning to the laboratory, were stored on ice in small coolers. In the laboratory, samples were placed in a refrigerator and held at 4°C until they could be centrifuged later that same day. Because we wanted to measure baseline levels of corticosterone, we avoided disturbing females immediately before capture by attaching ≈40 m of fishing line to the trap door early in the morning a capture was to be made. After a few hours had elapsed, the female was captured inside the nestbox by pulling on the fishing line to close the trap door. Captures occurred between 08:30–11:00 CDT. Because the lag time of the adrenocortical stress response has not been established for house wrens, we attempted to obtain a blood sample within three minutes of capture as recommended by Romero and Reed [43].The total time to obtain the blood sample (i.e., time from closing the trap door to the end of sample collection) varied from 105–270 s pre-supplementation (*n* = 71, mean ± SE = 153±4 s) to 89–209 s post-supplementation (*n* = 44, 147±4 s). After a blood sample was taken, we measured tarsus length (a measure of structural body size) to the nearest 0.1 mm with dial callipers and body mass to the nearest 0.1 g with a Pocket Pro scale (model # 250-B). If not already ringed, females were given a numbered, aluminium United States Geological Survey leg ring. After releasing the female at the second capture, the empty food canister and fishing line used to close the trap door were removed from the nestbox and the trap door secured in the open position.

### Laboratory procedures

On the day of collection, blood samples were centrifuged at 6000 rpm for 60 s to separate plasma from red blood cells (Hematastat II, Separation Technologies). Once the samples were centrifuged, haematocrit (% blood volume occupied by packed red blood cells) was determined as the mean of three readings, after which we recorded the plasma volume removed from the microcapillary tube with a 100-µL Hamilton syringe. All plasma was placed into individual locking-cap microcentrifuge tubes and stored at −20°C for around 4 months before further analysis.

An enzyme-linked immunoassay (corticosterone EIA) was performed on the stored plasma samples to determine plasma concentrations of corticosterone (Corticosterone EIA Kit, Cat. No. ADI-900-097; Enzo Life Sciences, Plymouth Meeting, PA). The assay procedure was conducted according to manufacturer instructions except we used the scaled-down, modified procedure of Wada et al. [44] because of our small plasma volumes. Three identical standards were run on each plate. All plasma samples and standards were run in duplicate to obtain a mean corticosterone concentration per sample. The plate was read on a microplate reader at 405 nm (corrected at 580 nm), and after the completion of the EIA, a 4-parameter logistic curve was created using Gen5 1.11.5 (BioTek Instruments, Inc., Winooski, VT) to obtain a standard curve. A conversion factor for each volume of plasma used per sample was applied in each calculation of final corticosterone concentration to account for the difference in actual volumes used in the assay (per well). Four plates were used in the analysis because of the large number of samples, but the inter-plate coefficient of variation (CV) remained low (5.4%) allowing for across-plate comparisons of results. In addition, the intra-plate CV for all four plates was within acceptable limits, ranging from 2.7% to 6.7% (mean ± SE = 4.3±0.9%).

Given baseline values reported for other species, and the fact that we detected no relationship between the time to obtain a blood sample and plasma corticosterone concentration (see Results), we are confident that we measured baseline corticosterone levels in incubating females and not merely handling-induced acute responses. Mean baseline values recorded for female house wrens in this study, 9.4 ng/mL in early incubation and 11.6 ng/mL shortly after hatching, fall well within the range of values recorded in other species [45], and are comparable to the mean value reported for four incubating female house wrens from another population (6.85 ng/mL; [46]). Although corticosterone values in female house wrens were highly variable, ranging from 2–50 ng/mL (SD = 10.0), this too is not unusual as similar variability in baseline values has been observed in other species [47], [48].

### Samples sizes and statistical analysis

By taking a pre- and post-supplementation blood sample, we could compare the change in plasma corticosterone levels using a repeated measures analysis, thus controlling for variation in corticosterone concentration among individual females. We obtained blood samples from 71 different females on incubation-day 2, of which 39 abandoned their nest after they had been captured and 32 did not. Of the 39 females that abandoned, 15 did not re-nest during the period of the first brood, 12 re-nested after abandoning but later abandoned their clutches after being captured on incubation-day 2 at their re-nest, and 12 did not abandon their re-nest and produced young in the first brood. This level of abandonment is not unusual, as house wrens are sensitive to capture at the time they begin incubation, which is why we normally capture females at least a week after incubation has begun. Of the 27 females that abandoned their nest after capture and failed to re-nest during the first brood, 20 re-nested later in the season, and so the majority of these females appeared to suffer no long-lasting ill effects of their early blood sampling. Of the 44 females from which we obtained blood samples pre- and post-supplementation, the nests of four of these were lost after hatching, presumably to predation, and thus our final sample consisted of 40 females, 19 control and 21 food-supplemented.

All statistical analyses were performed using SAS software (SAS 9.2, SAS Institute, Cary, NC, U.S.A.) and were two-tailed with α = 0.05. All means reported are least-squares means unless stated otherwise. Plasma corticosterone level was log-transformed to meet the assumptions of parametric tests. To confirm that females randomly assigned to treatments did not differ initially in body mass, tarsus length, or clutch size, we used analysis of covariance (ANCOVA) in PROC GLM with treatment as the main effect and clutch-initiation date as a covariate to control for any seasonal effect on mass and clutch size. Sample sizes of some measures for females are unequal because of missing values.

We used multivariate analysis of covariance (MANCOVA) in PROC GLM to assess the effects of treatment and clutch-initiation date on mean incubation constancy, mean on-bout length, mean off-bout length, and number of bouts per hour, after testing for equal slopes. The interaction between treatment and clutch-initiation date was not statistically significant, indicating slopes did not differ (*P*>0.05); hence, we omitted the interaction from the final model. We interpreted MANCOVA results using standardized canonical coefficients as described by Scheiner [49].

We used ANCOVA to determine if the plasma corticosterone level of females that abandoned their clutch after the pre-supplementation blood sample was taken was significantly different from females that did not abandon, including clutch-initiation date as a covariate. We employed linear regression to examine the effect of corticosterone level (both pre- and post-supplementation) separately on the four measures of incubation behaviour separately. We also used linear regression to explore the relationship between corticosterone level (both pre- and post-supplementation) and female condition, using residual mass based on a regression of log (mass) on (log) tarsus as a proxy for condition.

We employed three separate repeated-measures ANOVAs in PROC MIXED to examine the effect of food supplementation on female plasma corticosterone level, body mass, and haematocrit, with time (pre- and post-supplementation) as the repeated measure. We predicted that if food supplementation influences female condition, there should be a significant interaction between time and treatment. We initially included clutch size and clutch-initiation date as covariates in the models, but after finding that they had no appreciable effects on any aspect of female condition (results not shown), we omitted these variables from the final model.

## Results

### Pre-treatment comparisons

Prior to experimental food supplementation, ANCOVA showed no significant difference between treatments in female body mass (mean ± SE, control: 12.4±0.1 g, food-supplemented: 12.3±0.1 g; *F*_1,67_ = 0.37, *P* = 0.54) or tarsus length (control: 18.9±0.1 mm, food-supplemented: 18.8±0.1 mm; *F*_1,67_ = 0.41, *P* = 0.52); neither trait varied with clutch-initiation date (*P*>0.05 in both cases). There was also no significant difference between treatments in the mean clutch size of females (control: least-squares mean ± SE, 6.9±0.1 eggs, food-supplemented: 6.8±0.1 eggs; *F*_1,68_ = 0.43, *P* = 0.51); however, there was a significant effect of clutch-initiation date, with clutch size decreasing over the course of the season (parameter estimate  = −0.0326, *F*_1,68_ = 11.18, *P* = 0.0013).

### Female incubation behaviour

**Figure 2:**
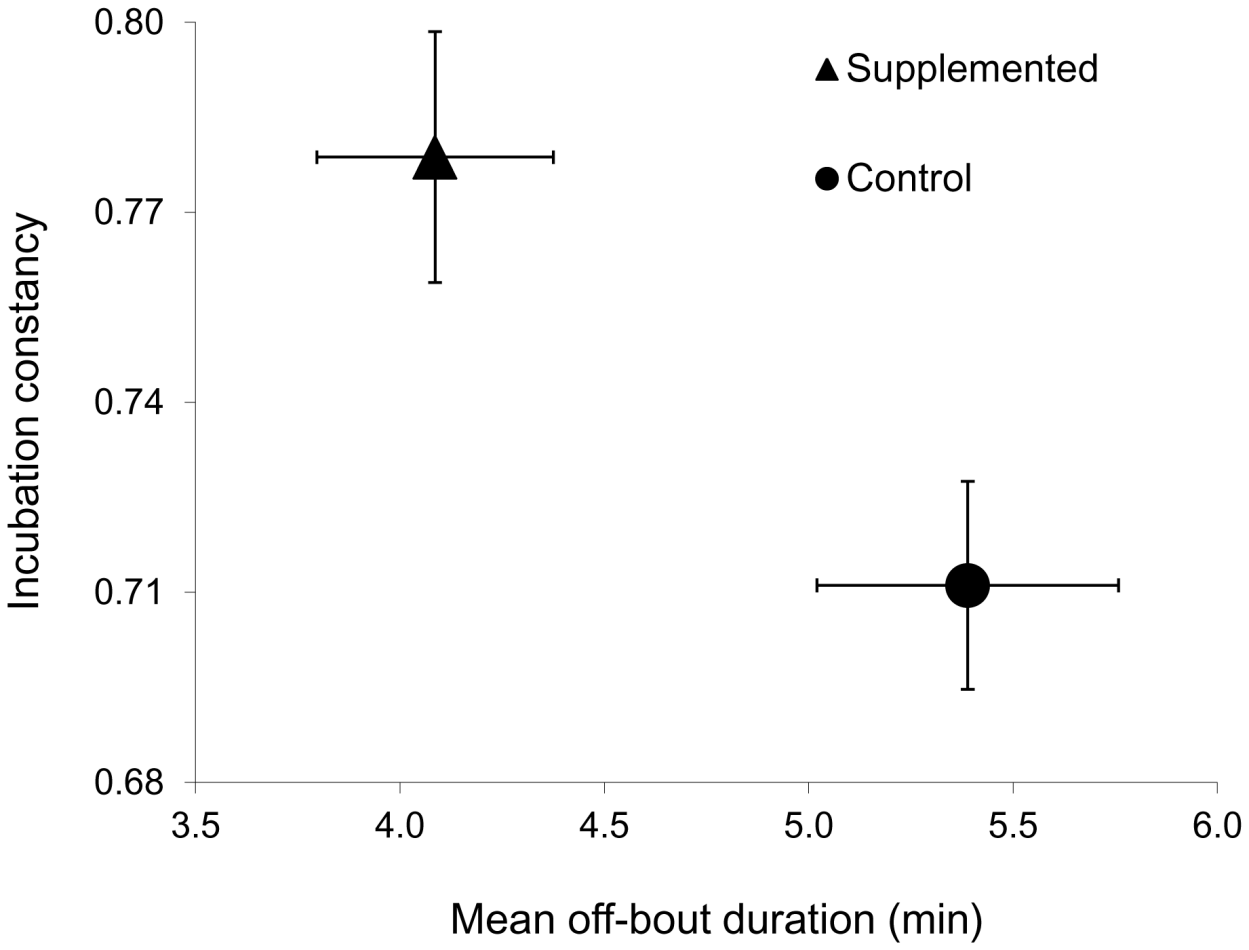
Bivariate means (±SE) of incubation constancy and mean off-bout length in experimentally supplemented and control female house wrens. Significant MANOVA effects are as described in Table 1.

MANCOVA revealed a significant effect of food supplementation on female incubation behaviour, but no effect of clutch-initiation date (Table 1). The magnitude of the standardized canonical coefficients showed that incubation constancy, on-bout length, and off-bout length contributed roughly the same to the significant treatment effect, whereas the number of incubation bouts contributed less so. These effects are illustrated in Fig. 2, which shows that supplemented females exhibited greater incubation constancy and shorter mean off-bout lengths than control females. Although food-supplemented females increased their nest attentiveness, the length of the incubation period did not differ significantly between treatments (*F*_1,41_ = 0.30, *P* = 0.36). However, there was a significant effect of clutch-initiation date on the length of the incubation period, with longer incubation periods occurring earlier in the season than later (parameter estimate  = −0.0425, *F*_1,41_ = 30.76, *P*<0.0001); the interaction between treatment and clutch-initiation date was not significant (*F*_1,41_ = 0.94, *P* = 0.34).

**Figure 3:**
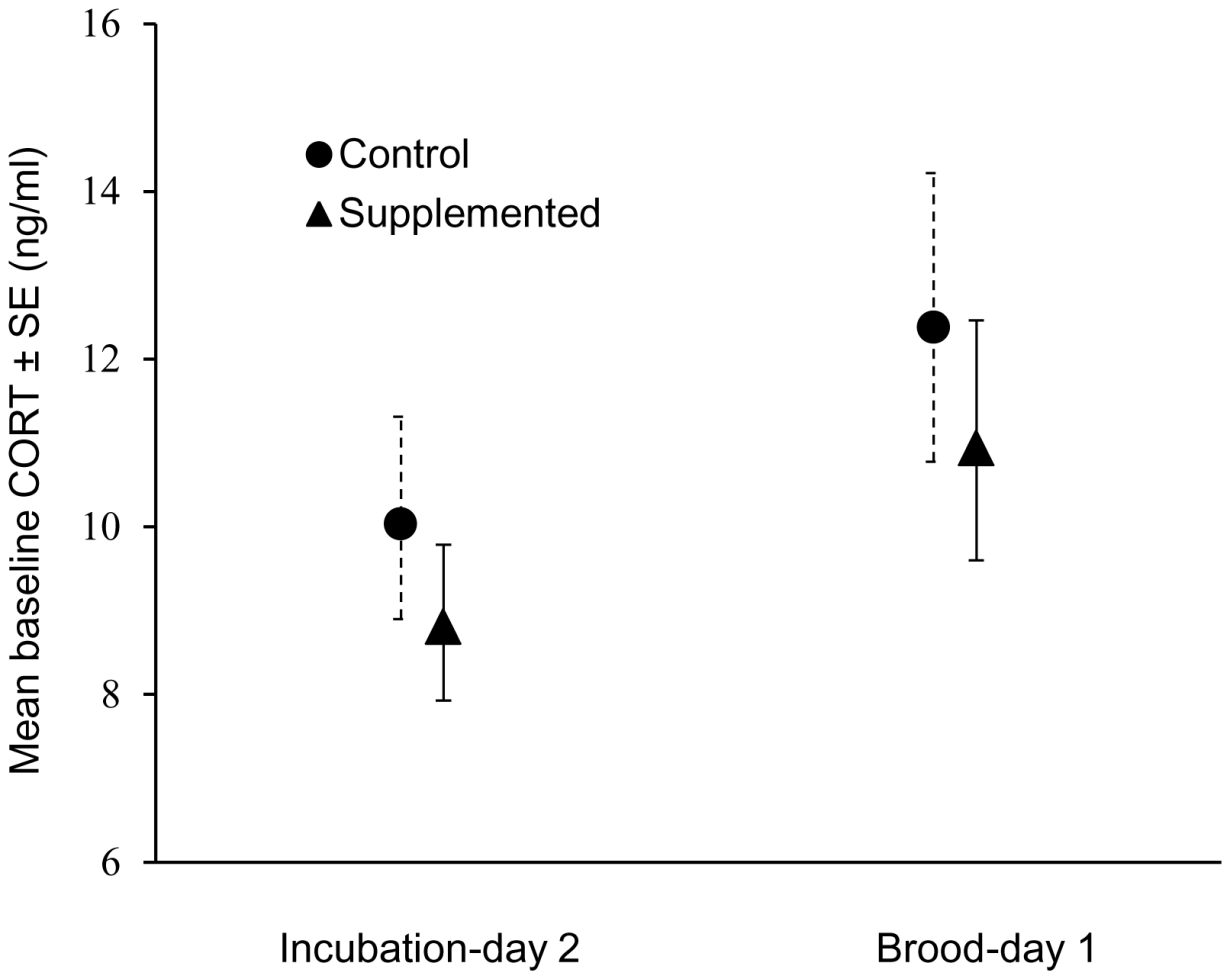
Repeated measures ANOVA of female baseline plasma corticosterone (ng/mL ± SE). Values in graph are from back-transformed least-squares means.

**Table 1:** MANCOVA of the effect of food supplementation and clutch-initiation date on mean incubation constancy, mean on-bout length, mean off-bout length, and number of bouts per hour.

				Standardized canonical coefficients			
Effect	Wilks' *λ*	*F_df_*	*P*	InConst	On-bout	Off-bout	Bouts/hr
Treatment	**0.6280**	**5.02_4,34_**	**0.0027**	**−1.0391**	**2.1507**	**−1.5163**	**0.6635**
Egg 1 Date	0.9751	0.22_4,34_	0.9271	1.4072	−0.6038	1.6763	1.6414

### Plasma corticosterone and nest abandonment, incubation, and food supplements

The time to obtain a blood sample and plasma corticosterone concentration were not correlated (linear regression pre-supplementation: *r^2^* = 0.005, *F*_1,69_ = 1.33, *P* = 0.25; post-supplementation: *r^2^* = −0.02 *F*_1,42_ = 0.32, *P* = 0.58). ANCOVA revealed that the mean corticosterone level (log-transformed) on incubation-day 2 was significantly higher in those females that abandoned their nest after blood sample collection (least-squares mean ± SE, 1.072±0.055 ng/mL) compared with those females that never abandoned their nest (0.921±0.043 ng/mL, *F*_1,68_ = 4.59, *P* = 0.0358); corticosterone level did not vary with clutch-initiation date (*F*_1,68_ = 2.76, *P* = 0.1015).

There were no significant linear relationships between female plasma corticosterone levels and incubation constancy, mean on-bout length, mean off-bout length, hatching success, fledging success, or clutch size (all *P*>0.05). Female corticosterone levels increased significantly over the course of the incubation period (*F*_1,48.6_ = 4.48, *P* = 0.04; Fig. 3), but food-supplemented and control females did not differ significantly in corticosterone levels (*F*_1,61.8_ = 0.40, *P* = 0.53).

There was no significant linear relationship between female condition and female plasma corticosterone level, either pre- (*r^2^* = 0.01, *F_1,68_* = 0.81, *P* = 0.37) or post-supplementation (*r^2^* = 0.01, *F_1,41_* = 0.30, *P* = 0.59).

### Female mass, haematocrit, and hatching success

Repeated-measures ANOVA showed no significant effect of treatment on female body mass (control: 12.0±0.1 g, food-supplemented: 11.9±0.1 g; *F*_1,67.6_ = 1.24, *P* = 0.27), nor was the time × treatment interaction significant (*F*_1,43.5_ = 1.73, *P* = 0.20). Female mass decreased significantly from incubation-day 2 (12.4±0.1 g) to brood-day 1(11.6±0.1 g) (*F*_1,44.7_ = 144.2, *P*<0.0001). Female haematocrit was not significantly altered by food supplementation (control: 47.1±0.6%, food-supplemented: 47.3±0.6%; *F*_1,62.5_ = 0.08, *P* = 0.78), nor did it change significantly from incubation-day 2 (46.7±0.5%) to brood-day 1: 47.8±0.6%) (*F*_1,54.1_ = 2.21, *P* = 0.14). The time × treatment interaction was also not significant (*F*_1,54.1_ = 0.03, *P* = 0.87).

ANCOVA showed no significant difference in the hatching success of eggs laid by control females (96.0±3.4%) and those laid by food-supplemented females (88.7±3.2%) (*F*_1,37_ = 2.35, *P* = 0.1338); hatching success was not affected by clutch-initiation date (*F*_1,37_ = 0.00, *P* = 0.9975). Food supplementation had no effect on whether or not a female produced a second brood after her successful first brood (percentage double-brooded, control: 61.1±4.8%, food-supplemented: 65.0±6.2%; χ^2^ = 0.06, *P* = 0.80).

## Discussion

Food-supplemented females spent a greater proportion of their time on the nest and spent less time away from their eggs, presumably foraging for food, than control females. This change in female incubation behavior is consistent with the hypothesis that the availability of food determines how much time females can spend on the nest. When females leave their nest to forage, egg temperatures begin to drop, and they must soon return to the eggs to ensure they do not cool to temperatures that harm or even kill the developing embryo [50], [51]. Because food-supplemented females spent more time incubating their eggs than controls, incubating females under normal circumstances might experience a negative energy balance that prevents them from investing more time and energy into incubation effort. However, there was no evidence in this study that increased incubation constancy resulted in benefits for the females as food supplementation had no significant effect on female mass, haematocrit, baseline plasma corticosterone levels, or reproductive success.

Although food-supplemented female house wrens increased their incubation constancy and averaged shorter off-bouts, they did not have significantly lower baseline corticosterone levels than control females shortly after hatching began. This result is contrary to the expectation that females that are less food limited than others should have lower corticosterone levels [32]. However, females in both treatment groups did experience an increase in baseline corticosterone levels between the first to the second sample periods.

One explanation for this change could be that it reflects a gradual increase in baseline corticosterone levels associated with the demands of incubation. Although this may explain some of the increase, we think that the increase more likely occurred shortly before hatching and reflected changes associated with the beginning of the highly demanding nestling provisioning stage [52]-[55]. The second measurement of corticosterone levels was taken shortly after hatching began and not late in the incubation period, so this significant increase in baseline plasma corticosterone levels over that found during early incubation may stem from a ‘preparative’ response to a forthcoming increase in foraging behaviour to provide food for the hatchlings (discussed in [56], p. 239). In house wrens, there is a marked decline in body mass between the last days of the incubation period and the first few days of the nestling period that is interpreted as an adaptation to reduce wing-loading during the nestling-provisioning period [37]. As it is well established that glucocorticoids mediate the mobilization of energy from the breakdown of body tissues (reviewed in [30]), the increased plasma corticosterone level found shortly after the onset of hatching that we report here may be playing a preparative role for the demands of nestling provisioning, which includes reducing wing-loading. Indeed, Bonier et al. [54] found that increases in female baseline corticosterone can be beneficial during nestling provisioning, with intermediate levels of corticosterone (i.e., levels below those produced by acute stress) leading to increased reproductive success through increased provisioning rates. Similarly, Ouyang et al. [57] found that an experimental increase in corticosterone within the natural range of variation resulted in increased reproductive effort during the incubation stage in great tits (*Parus major*).

In addition to the change in incubation behaviour of food-supplemented females, perhaps the best evidence from this study supporting the supposition that incubation can be demanding, at least for some females, is that those females that abandoned their clutches after the first blood sample was taken had significantly higher baseline plasma corticosterone concentrations than those that did not abandon their clutches. This suggests that there is an individual threshold level of baseline corticosterone, above which females that experience a stressful situation (e.g., capture and blood-sampling) will abandon their reproductive attempt. In line with this hypothesis, Ouyang et al. [58] found that house sparrows (*Passer domesticus*) with low pre-breeding but high breeding corticosterone levels produced more fledglings, which suggests that individuals with low pre-breeding corticosterone levels were more likely to initiate clutches and that corticosterone is subsequently up-regulated to meet the challenge of provisioning a brood. As in house wrens, European starlings (*Sturnus vulgaris*) that abandoned their nests after capture had higher levels of baseline corticosterone than those that did not [52]. The observation that high baseline corticosterone levels are associated with nest abandonment may provide an answer to the question, posed by Monaghan and Spencer [59], of whether comparatively high levels of plasma corticosterone are indicative of individuals failing or succeeding to meet the challenges presented by their environment. In the cases of nest abandonment by house wrens, the former seems more likely than the latter. However, Criscuolo et al. [60] argue that elevated corticosterone levels alone do not lead to nest desertion. In their study, although female common eiders (*Somateria mollissima*) with corticosterone implants had a significant increase in plasma corticosterone, a decrease in prolactin levels (a hormone linked to parental care), and a steady decrease in mass, they did not abandon their nests during incubation. At least in capital breeders (i.e., species, such as eiders, that rely on energy reserves to compensate for high metabolic demands during and after incubation [61]), corticosterone concentrations must be elevated, prolactin levels must reach a ‘threshold low value,’ and use of proteins as an energy source must increase for abandonment to occur [60]. However, it is unknown whether these same criteria apply to income breeders, such as house wrens, that do not rely on large, stored energy reserves during incubation.

The lack of a significant treatment effect on female plasma corticosterone levels, body mass, and haematocrit could have been caused, at least in part, by a reduction in the range of differences in female quality brought about by the experimental protocol. The females in the study that did not abandon their nests after their first capture were only those that fell within the lower range of baseline corticosterone levels. Females falling within the upper range of baseline corticosterone levels at first capture might have been unable to tolerate any further strain and abandoned reproduction in favour of self-maintenance. This is consistent with the observation that elevation of plasma corticosterone is associated with inhibition of egg production (reviewed in [56]). It is possible that female house wrens that abandoned their nests after being captured were of low quality or young, inexperienced females, as has been reported in other species [62]. Had these females not been captured and bled, they probably would have continued to nest. However, by losing these females from the experiment, detecting an effect of the treatment was probably less likely because average and above-average quality females would not be as affected by the presence of additional food as would low-quality females (cf., [63]). This may simply be because weather conditions during the time of the study were close to the average of the preceding 10 years, as was reproductive success. Perhaps these ‘average’ conditions and an apparently sufficient food supply, as indicated by ‘average’ reproductive success, created a sufficiently benign environment so that there were no detectable effects of increased food availability on these females of average and above-average quality.

In conclusion, food-supplemented females devoted more time to incubating their eggs than control females, but this did not significantly affect the length of their incubation periods. Although plasma corticosterone levels increased significantly between the beginning of incubation and shortly after hatching began in both supplemented and control females, supplemented females did not have lower baseline corticosterone levels than controls after the incubation period. Supplemented and control females also did not differ in body mass, haematocrit, or reproductive success. This lack of difference in physiological traits and reproductive success may have come about because only those food-supplemented and control females with low baseline corticosterone levels early in the incubation period (i.e., likely high-quality females) did not abandon their clutches after the first blood sample and thus remained to be sampled after hatching had begun. We suggest that under natural conditions, those females with high baseline plasma corticosterone levels are more likely than those with low levels to abandon their reproductive attempt after exposure to stressful situations.
